# Profils épidémiologiques des acidocétoses diabétiques aux urgences

**DOI:** 10.11604/pamj.2019.33.322.17161

**Published:** 2019-08-26

**Authors:** Sarra Jouini, Asma Aloui, Olfa Slimani, Fatma Hebaieb, Rym Ben Kaddour, Héla Manai, Hana Hedhli

**Affiliations:** 1 Hôpital Charles Nicolle, Service des Urgences, Tunis, Tunisie; 2 Université Tunis El Manar, Faculté de Médecine de Tunis, Tunis, Tunisie; 3 Hôpital Charles Nicolle, Service de Gynécologie Obstétrique, Tunis, Tunisie

**Keywords:** Acidocétose diabétique, urgence, clinique, traitement, Diabetic ketoacidosis, emergency department, clinical, treatment

## Abstract

**Introduction:**

L'acidocétose diabétique (ACD) est une complication métabolique grave du diabète. Son incidence est en augmentation ces dernières années, cependant sa mortalité reste faible. L'objectif de cette étude a été de décrire les caractéristiques épidémiologiques, cliniques, thérapeutiques et pronostiques des patients admis aux urgences pour ACD sévère ou modérée.

**Méthodes:**

Il s'agissait d'une étude prospective, descriptive qui a inclus les ACD modérées ou sévères. Standardisation du protocole de prise en charge thérapeutique. Nous avons étudié les caractéristiques épidémiologiques, cliniques, thérapeutiques et pronostiques chez ces patients.

**Résultats:**

Nous avons inclus 185 patients avec ACD sévère ou modérée. L'âge moyen a été de 38 +/- 18 ans; le sexe ratio=0,94. Diabète connue= 159 patients (85%) dont 116 étaient des diabétiques type 1. Les facteurs de décompensation les plus fréquents étaient l'arrêt du traitement chez 42% et l'infection chez 32%. La glycémie moyenne a été de 32,7+/-12mmol/L, pH =7,14+/-0,13, HCO3- =7,2+/-3,56 mmol /L. La durée moyenne de l'insuline intraveineuse était de 17,3 +/- 16 heures. L'hypoglycémie a été observée chez 26 patients (14%), l'hypokaliémie chez 80 (43%). La mortalité au cours de l'hospitalisation a été de 2,1%.

**Conclusion:**

L'acidocétose diabétique survient chez les sujets jeunes traités par insulinothérapie. Le traitement est à base d'insuline par voie intraveineuse en plus de la correction du déficit hydrique. Les complications sont essentiellement l'hypokaliémie et l'hypoglycémie; et la mortalité reste faible.

## Abstract (en)

**Introduction:**

Diabetic ketoacidosis (DKA) is a severe metabolic complication of diabetes. Recent years have seen a marked increase in prevalence of diabetic ketoacidosis, but mortality is low. This study aimed to describe the epidemiological, clinical, therapeutic and prognostic features of patients with severe or moderate DKA admitted to the Emergency Department.

**Methods:**

He conducted a prospective, descriptive study including patients with moderate or severe DKA. Standardized care protocol. We studied the epidemiological, clinical, therapeutic and prognostic features of these patients.

**Results:**

The study involved 185 patients with moderate or severe DKA. The average age of patients was 38+/-18 years, with a sex ratio of 0.94. Known diabetes was reported in 159 patients (85%) of whom 116 had type 1 diabetes. The most common factors of decompensation were treatment discontinuation in 42% and infection in 32%. Average blood glucose was 32.7+/-12 mmol/L, pH =7.14+/-0.13, HCO3- =7.2+/-3.56 mmol/L. The mean duration of intravenous insulin was 17.3 +/- 16 hours. Hypoglycaemia was reported in 26 patients (14%), hypokalemia in 80 (43%) patients and hyperchloraemic mineral acidosis in 43 patients (23%). Intrahospital mortality was 2.1%.

**Conclusion:**

Diabetic ketoacidosis occurs in young subjects treated with insulin therapy. Treatment is based on intravenous insulin associated with correction of fluid deficit. Complications mainly include hypokalemia and hypoglycemia and mortality is low.

## Introduction

L'acidocétose diabétique (ACD) représente une complication aiguë grave du diabète [1]. Avec l'augmentation de la prévalence du diabète dans le monde, ce déséquilibre glycémique représente l'un des principaux motifs d'admission aux urgences [1,2]. Sa physiopathologie repose essentiellement sur une insulinopénie absolue ou relative ou un état d'insulinorésistance et/ou une augmentation des hormones de contre-régulation [2,3]. Cette situation est responsable d'une accumulation de corps cétoniques, une déplétion hydrique associée à des désordres électrolytiques [2,3]. La découverte de l'insuline par Dr Frederik Banting en 1921 a transformé le pronostic de ce trouble métabolique potentiellement grave [1,4]. Cette complication était grevée d'une lourde mortalité en début du vingtième siècle et qui a diminué à moins de 5% durant les dernières décennies [1,5-7]. Cela est dû à la standardisation du protocole de la prise en charge de l'ACD qui repose essentiellement sur trois volets la correction de la déshydratation et la restauration de la volémie par l'apport des cristalloïdes; l'insulinothérapie par la voie intraveineuse et la correction des troubles électrolytiques en plus du contrôle du facteur de décompensation [2]. L'objectif de cette étude était de décrire les caractéristiques épidémiologiques, cliniques, thérapeutiques et pronostiques des patients admis aux urgences pour ACD sévère ou modérée.

## Méthodes

Il s'agissait d'une étude monocentrique, prospective descriptive. Notre travail a été mené au service des urgences de l'hôpital Charles Nicolle à Tunis, Tunisie; sur une période étendue de mai 2015 au novembre 201^6^; incluant 185 patients admis pour acidocétose diabétique modérée ou sévère. Nous avons inclus les patients âgés de plus de 16 ans qui s'étaient successivement présentés aux urgences dans un tableau d'ACD modérée à sévère selon les critères de l'American Diabetes Association (ADA) (Tableau 1) [2]. L'ACD modérée à sévère est définie par un taux de bicarbonates <15 mmol/L et/ou un pH sanguin ≤7,24. Ont été également inclus les ACD avec un pH entre 7,25 et 7,35 en cas d'association de l'ACD à une alcalose respiratoire définie par une PaCO2 mesurée < PaCO2 attendue selon la formule: PaCO2 attendue = 1,5 × HCO3- (mmol/L) + 8 ± 2, n'ont pas été inclus les patients âgés de moins de 16 ans ou avec une kaliémie initiale inférieur à 3,3 mmol /L. Ont été exclus les patients avec une insuffisance rénale chronique connue empêchant l'imputation de l'acidose métabolique à la seule ACD; et les décompensations hyperglycémies-hyperosmolaires. Tous les patients ont été hospitalisés au niveau du service des urgences initialement à l'unité de surveillance rapprochée (USR) puis à l'unité d'hospitalisation de courte durée (UHCD) une fois leur état clinique est stabilisé. Le protocole thérapeutique a été standardisé et a reposé sur plusieurs volets:

**Tableau 1:** Classification de l'acidocétose diabétique selon l’American Diabetes Association [2]

	Acidocétose diabétique		
	Légère	Modérée	Sévère
Glycémie (mmol/L)	>14	>14	>14
pH sanguine	7,25-7,30	7,0-7,24	<7
Trou anionique (mEq /L)	>10	>12	>12
Bicarbonatémie (mmol/L)	15-18	10<15	<10
Cétonurie	Positive	positive	positive

***La réhydratation***: avec estimation des pertes hydriques à 10% du poids corporel. Restitution du déficit sur 24 heures par des cristalloïdes type sérum salé isotonique à 0,9% (NaCl 0,9%) seul si glycémie capillaire (GC) >2,5g/L et sérum glucosé à 5% (SG5%) en dérivation avec le Na Cl 0,9% si la GC <2,5g/L.

L'insulinothérapie par voie intraveineuse continue avec l'apport de l'insuline ordinaire à la dose de 0,1U/kg/heure en intraveineux (IV) continu au pousse seringue électrique moyennant une surveillance horaire de la glycémie capillaire. Les critères de transition de l'insuline IV à l'insuline en sous-cutané (S/C) ont été standardisés avec l'obtention d'une glycémie capillaire <2,5 g/L associée à deux critères ou plus parmi les suivants: pH> 7,3, HCO3- > 18mmol/L, Trou anionique <12mEq/L - La supplémentation potassique moyennant une évaluation continue de la fonction rénale et de la diurèse et une surveillance de la kaliémie sur les gaz du sang. -Une prise en charge spécifique du facteur de décompensation a été réalisée en parallèle. On a procédé à une étude des caractéristiques épidémiologiques, cliniques, thérapeutiques et pronostiques chez ces patients.

Analyse statistique: l'acquisition des données et l'étude statistique ont été réalisées au moyen du logiciel SPSS 19.0 for Windows. Nous avons mené une étude descriptive: avec calcul des fréquences simples et des fréquences relatives pour les variables qualitatives. Calcul des moyennes, des médianes, des écarts-types, et de l'étendue pour les variables quantitatives.

Aspects éthiques: nous avons mené une étude observationnelle descriptive; aucune intervention à visée exploratrice ou thérapeutique n'a été imposée. Le protocole thérapeutique exposé a été celui du service.

## Résultats

**Figure 1:**
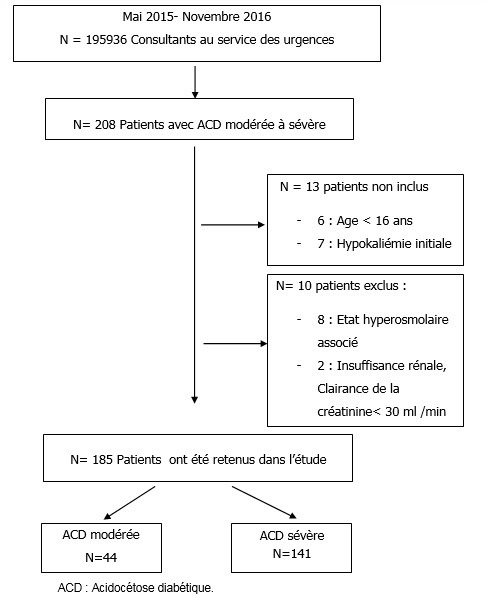
Diagramme de flux des patients

Durant la période mai 2015 - novembre 2016, 185 patients avec acidocétose diabétique ont été retenus dans l'étude; 141 avec ACD sévère et 44 patients avec ACD modérée selon la classification de l'ADA. La figure 1 schématise le diagramme de flux des patients inclus dans l'étude. La moyenne d'âge a été de 38 +/- 18 ans avec des extrêmes allant de 16 à 85 ans. Cent vingt un patients (65%) étaient âgés de moins de 45 ans. Le sex ratio était de 0,94. Le Tableau 2 résume les antécédents des patients. Les signes fonctionnels étaient essentiellement type nausée-vomissement chez 130 patients soit 70%; douleurs abdominales chez 63 (34%) et syndrome polyuropolydipsique chez 46 patients soit 25%. Deux patients avaient une altération profonde de l'état de conscience avec un Glasgow Coma Scale (GCS) initial ≤8; 161 patients (87%) avaient un état de conscience parfaitement conservé avec GCS à 15; le reste des patients avaient une altération légère à modérée de l'état de conscience. Le Tableau 3 résume les paramètres cliniques, gazométrique et biologiques de la population. L'arrêt du traitement et l'infection représentaient les causes de décompensation les plus fréquentes dans cette série retrouvées respectivement chez 42% et 32% des patients. Les sites infectieux les plus fréquents étaient: la sphère otorhinolaryngologie dans 25 % des cas, urinaire dans 23% et pulmonaire dans 14,5% des cas. Sur le plan thérapeutique la durée moyenne de l'insulinothérapie intraveineuse a été de 17,3 +/- 16 heures avec des extrêmes allant de 4 à 144 heures. Les effets secondaires enregistrés étaient essentiellement type hypokaliémie et hypoglycémie en plus de la survenue d'acidose minérale hyperchlorémique (Tableau 4). La majorité des patients ont été pris en charge totalement au niveau du service des urgences; 16 patients ont été transférés vers les services d'endocrinologie ou de médecine interne, 8 patients vers des services de chirurgie et un seul patient a été admis dans un service de réanimation. La durée moyenne d'hospitalisation au niveau des services des urgences a été de 61+/- 42 heures répartit entre l'USR avec une moyenne de 30 +/-21 heures et l'UHCD de 32 +/- 28 heures. Quatre décès ont été enregistrés avec une mortalité intrahospitalière de 2,1% secondaire à un état de choc septique.

**Tableau 2:** Antécédents et traitements antérieurs des patients

Caractéristiques	Echantillon (N=185)
**Antécédents N (%)**	
Diabète connue	159 (85)
Diabète type 1	116 (62,4)
Diabète type 2	15 (8,1)
Diabète insulino-nécessitant	28 (15)
ACD Inaugurale	26 (14)
Hypertension artérielle	29 (15,6)
Dyslipidémie	9 (4,8)
**Traitement antérieur N (%)**	
Insuline	144 (77)
Biguanide	16 (8)
Sulfamide	4 (2)

ACD: acidocétose diabétiques

**Tableau 3:** Paramètres cliniques, gazométriques et biologiques des patients

Variables	Moyenne ± ET	Valeurs extrêmes
**Paramètres cliniques**		
PAS (mmHg)	147±15	80-180
PAD (mmHg)	78±12	40-110
FC (battements /min)	102±16	60-148
FR (cycles/min)	28 ±6	16-44
SpO_2_ Air ambiant (%)	98±3	70-100
Température (°C)	37,3±0,6	36-39,9
BMI (kg/m^2^)	25±3	18,9-31
**Paramètres gazométriques**		
pH	7,14±0,13	6,80-7,35
HCO_3_^-^	7,2±3,56	3-17,6
**Paramètres biologiques**		
Glycémie (mmol/L)	32,7±12	15 -74,4
Créatinine (μmol/L)	146±83	56- 746
Trou anionique	29±6,5	13-49

**BMI**: body mass index; **ET**: écart type; **FR**: fréquence respiratoire; **FC**: fréquence cardiaque; **PAS**: pression artérielle systolique; **PAD**: pression artérielle diastolique; **SpO_2_**: saturation pulsée en oxygène

**Tableau 4:** Tolérance et effets secondaires du traitement de l’acidocétose diabétique

	N (%)
Hypokaliémie <3,3 mmol/L	80 (43)
Hypoglycémie < 0,5 g/L	26 (14)
Acidose minérale hyperchlorémique	43 (23)

## Discussion

Notre étude a montré que l'acidocétose diabétique survient essentiellement chez les sujets jeunes traités par l'insuline. Le traitement est à base d'insuline par voie intraveineuse en plus de la correction du déficit hydrique. Les complications sont essentiellement l'hypokaliémie et l'hypoglycémie; et la mortalité reste faible. Cette étude présente beaucoup de points forts notamment l'aspect prospective, l'inclusion d'un échantillon large de patients par rapport aux études de la littérature, l'exclusion des patients avec un tableau intriqué: ACD associée à un état hyperosmolaire. En plus notre étude a été faite dans un service d'Urgence Médico-chirurgicale Polyvalent. Les conditions d'hospitalisation, de surveillance et de monitorage des malades reflètent mieux la réalité par rapport à plusieurs études de la littérature qui ont été réalisées dans des laboratoires de recherche. Les services d'urgence polyvalents, représentent actuellement le lieu de prise en charge et d'hospitalisation des patients en ACD dans notre pays. Bien que l'aspect monocentrique et observationnel descriptif reste la limite la plus importante de l'étude; l'absence d'autres paramètres tels que le dosage de la cétonémie, paramètre important pour l'évaluation de la sévérité du tableau initial d'ACD et de l'évolution sous traitement représente aussi une limite de l'étude. Le diabète est un problème majeur de santé publique à l'échelle mondiale [8,9]. En 2010, plus que 285 millions d’adultes ont été atteints de diabète avec des dépenses estimées à plus que 376 billions de dollars par an dans le monde [8,10]. Son incidence augmente régulièrement avec un âge de survenue de plus en plus jeune, ce qui expose à plus de complications aiguës et chroniques [8,9,11]. Les complications aiguës du diabète représentent un motif de consultation et d'hospitalisation fréquent dans les services d'urgence [9,12].

L'ACD est considérée comme une complication aiguë sévère du diabète, vu l'importance de sa morbi-mortalité [5,13]. Son incidence annuelle aux États-Unis d'Amérique (USA) est estimée entre 4,6 et 8 épisodes/1000 diabétiques avec une constante augmentation [1,9,11,14]. Cette incidence est encore plus importante dans d'autres pays notamment en Bretagne, en Suède et dans les pays en voie de développement [1]. Les caractéristiques démographiques et les antécédentes des patients inclus dans les études de la littérature s'approchent de ceux de nos patients avec un âge moyen entre 28 et 41 ans et une prédominance des diabétiques type 1 [15,16]. L'arrêt de l'insulinothérapie et l'infection représentaient les facteurs de décompensation les plus fréquents dans notre étude ce qui concorde avec la littérature récente [17-19]. Les dernières décennies ont vu un développement important dans la prise en charge de l'ACD avec une standardisation du protocole de prise en charge ce qui a permis la diminution de sa morbi-mortalité, d'améliorer le pronostic et de raccourcir la durée d'hospitalisation [6,7]. L'insulinothérapie reste une pierre angulaire dans le traitement de l'ACD vu qu'elle présente la seule étape capable d'inhiber la cétogenèse et contrarier les effets des hormones de contre-régulation permettant ainsi la correction de l'acidose métabolique organique. Son utilisation par voie intraveineuse continue dans les ACD modérées à sévères a fait l'objet de plusieurs études cliniques et reste la voie actuellement recommandée par l'ADA [2,15,16,18]. Les doses recommandées ont varié de 0,1 à 0,14 U/Kg/H avec ou sans bolus intraveineux direct initial. Dans notre étude l'insulinothérapie a été utilisée par voie intraveineuse à la dose de 0,1 U/Kg/H sans bolus initial. La réhydratation constitue aussi le premier volet thérapeutique de l'ACD. Elle permet à elle seule en plus de l'apport hydrique, d'améliorer la fonction rénale de réduire l'insulinorésistance en diminuant la sécrétion des hormones de contre régulation dont la sécrétion augmente dans les états hyperglycémiques [20]. Le sérum salé isotonique, soluté de réhydratation de référence, a été utilisé dans cette étude avec adjonction du sérum glucosé à 5% pour des valeurs de glycémie inférieur à 2,5g/L afin de continuer l'administration d'insuline jusqu'à la disparition de l'acidose et d'éviter l'hypoglycémie [21].

Dans notre étude, les complications type hypoglycémie et hypokaliémie étaient les plus fréquentes. Dans la littérature, l'hypoglycémie a été rapportée dans 5-25% des cas, ceci est similaire aux résultats observés dans notre étude [2,22,23]. Le taux de survenue d'hypoglycémie dans notre étude a été de 14%; ceci peut être expliqué en partie par la diminution de la surveillance (la majorité des épisodes d'hypoglycémie ont été enregistrés au cours de la garde de nuit). Cependant, la survenue de l'hypoglycémie en tête de liste des complications du traitement de l'ACD dans la littérature a été remplacée dans notre étude par l'hypokaliémie dont la fréquence était plus élevée. L'ACD est associée à un déficit potassique qui peut être masqué voire même remplacé initialement par une hyperkaliémie à cause de l'acidose, de la proteïnolyse et de l'insulinopénie [2,3,24]. La correction de ces troubles par l'apport hydrique et l'insulinothérapie peut démasquer ce déficit. Pour pallier à cette complication, l'apport en potassium doit être considéré au-dessous d'une valeur de 5-5,3 mEq/L chez les patients avec fonction rénale normale à raison de 20-30 mEq/L afin de maintenir une kaliémie entre 4 et 5 mEq/L [2,3,24]. Ces recommandations ont été considérées dans notre étude mais non appliquées d'une manière stricte d'une part par faute d'un débimètre permettant de répartir de façon adéquate les apports en potassium et de garantir ainsi sa perfusion sur une durée bien précisée et d'autre part, par retard du bilan biologique à l'origine d'un retard de modification appropriée des apports en potassium. De même cette incidence élevée d'hypokaliémie peut être expliquée par la différence de la taille des échantillons par rapport aux études précédentes. La durée d'hospitalisation dans notre étude était plus courte par rapport à ce qui a été rapporté dans la littérature [15,16,23]. Ceci peut s'expliquer par le déroulement de l'étude dans un service des urgences recrutant un grand nombre de malades et dont la durée d'hospitalisation a couvert la durée de résolution de l'ACD. Les malades ont été orientés par la suite de façon différée vers des services spécialisés pour équilibration de leur diabète.

La mortalité décrite dans la littérature était < 2% des cas dans les pays développés [1,5,6]. Elle est secondaire au facteur de décompensation, dépend du terrain et se voit principalement aux âges extrêmes. Notre série a enregistré une mortalité de 2,1%. L'acidocétose diabétique relève toujours du domaine de la recherche médicale notamment le volet thérapeutique comme la nature du meilleur soluté permettant de restaurer l'hydratation au prix d'un faible taux d'effets secondaires et les modalités d'insulinothérapie en ce qui concerne la dose et la voie d'administration.

## Conclusion

L'acidocétose diabétique, complication aiguë et grave du diabète est fréquemment observée et prise en charge au niveau des services des urgences. Le traitement de l'ACD repose essentiellement sur trois volets: hydratation, insulinothérapie et correction des troubles électrolytiques. La standardisation des protocoles de prise en charge permet de diminuer la morbi-mortalité de cette complication et de diminuer les durées d'hospitalisation des patients.

### État des connaissances actuelles sur le sujet

- L'acidocétose diabétique est une complication aiguë et grave du diabète;
- L'apanage de sujet jeunes diabétiques insulinodépendants;
- Sa prise en charge repose essentiellement sur trois volets la correction de la déshydratation et la restauration de la volémie par l'apport des cristalloïdes; l'insulinothérapie et la correction des troubles électrolytiques en plus du contrôle du facteur de décompensation.

### Contribution de notre étude à la connaissance

- L'utilisation de protocole standardisé de prise en charge thérapeutique dans l'acidocétose diabétique permet de mieux contrôler la morbi-mortalité de cette complication et de diminuer la durée d’hospitalisation et donc forcément le coût;
- L'insulinothérapie par voie intraveineuse moyennant une surveillance adéquate utilisée dans l'acidocétose diabétique a un bon rapport efficacité innocuité.

## Conflits d’intérêts

Les auteurs ne déclarent aucun conflit d’intérêts.
